# Transcriptome analysis of critical genes related to flowering in *Mikania micrantha* at different altitudes provides insights for a potential control

**DOI:** 10.1186/s12864-023-09108-8

**Published:** 2023-01-10

**Authors:** Chen Liang, Ling Liu, Zhixiao Zhang, Sangzi Ze, Ling Pei, Lichen Feng, Mei Ji, Bin Yang, Ning Zhao

**Affiliations:** 1 College of Life Sciences, Southwest Forestry University, Kunming, 650224 China; 2 Yunnan Academy of Forestry and Grassland, Kunming, 650201 China; 3 Yunnan Forestry and Grassland Pest Control and Quarantine Bureau, Kunming, 650051 China; 4 Key Laboratory of Forest Disaster Warning and Control of Yunnan Province, Southwest Forestry University, Kunming, 650224 China

**Keywords:** *Mikania micrantha*, Transcriptome, Flower bud differentiation, Flowering regulatory pathway, Phytohormone, Differentially expressed gene

## Abstract

**Background:**

*Mikania micrantha* is a vine with strong invasion ability, and its strong sexual reproduction ability is not only the main factor of harm, but also a serious obstacle to control. *M. micrantha* spreads mainly through seed production. Therefore, inhibiting the flowering and seed production of *M. micrantha* is an effective strategy to prevent from continuing to spread.

**Result:**

The flowering number of *M. micrantha* is different at different altitudes. A total of 67.01 Gb of clean data were obtained from nine cDNA libraries, and more than 83.47% of the clean reads were mapped to the reference genome. In total, 5878 and 7686 significantly differentially expressed genes (DEGs) were found in E2 vs. E9 and E13 vs. E9, respectively. Based on the background annotation and gene expression, some candidate genes related to the flowering pathway were initially screened, and their expression levels in the three different altitudes in flower bud differentiation showed the same trend. That is, at an altitude of 1300 m, the flower integration gene and flower meristem gene were downregulated (such as SOC1 and AP1), and the flowering inhibition gene was upregulated (such as FRI and SVP). Additionally, the results showed that there were many DEGs involved in the hormone signal transduction pathway in the flower bud differentiation of *M. micrantha* at different altitudes.

**Conclusions:**

Our results provide abundant sequence resources for clarifying the underlying mechanisms of flower bud differentiation and mining the key factors inhibiting the flowering and seed production of *M. micrantha* to provide technical support for the discovery of an efficient control method.

**Supplementary Information:**

The online version contains supplementary material available at 10.1186/s12864-023-09108-8.

## Background

*Mikania micrantha* Kunth (the Asteraceae family) is an extremely pernicious vine and is one of the top 100 worst invasive alien species in the world [1]. As a noxious invasive plant, *M. micrantha* has caused enormous economic losses and ecological damage [2, 3]. With the in-depth study of *M. micrantha*, chemical control, physical control, and biological control have been used to control it, but they have not been prevented fundamentally [4–6]. The basic reason for this was that *M. micrantha* has a large number of flowers, and its flower biomass accounts for 38.4% ~ 42.8% of the total biomass [7]. *M. micrantha* is also called the Mile- A- minute weed, which vividly compares the rapid growth and diffusion of *M. micrantha* [8]. *M. micrantha* can grow and reproduce rapidly in a short time under asexual and sexual reproduction. The seed (sexual reproduction) is small and light in weight. However, mature seeds spread mainly through artificial and natural (such as wind or water) methods [9]. Inhibiting the sexual propagation of *M. micrantha* is an effective means to control its rapid spread. Therefore, regulating the flowering and seed production of *M. micrantha* is of great significance for defense and management. Through the division of the suitable areas of *M. micrantha* in Yunnan Province, He et al. found that *M. micrantha* in Dehong Prefecture is obviously distributed according to altitude. They also found that the damage area is the largest in the altitude range of 801–1000 m, and the number of flowers is the largest in the altitude area of 900 m [10]. In addition, another previous study found that with increasing altitude, the harmful area of *M. micrantha* decreased and the number of *M. micrantha* flowers also decreased. The area below 1100 m above sea level is the high-level suitable area of *M. micrantha*; when the altitude exceeds 1100 m, the occurrence probability of *M. micrantha* gradually decreases, which is an unsuitable area [11].

Flower bud differentiation is the most critical stage in the development of angiosperms. It marks the transformation from vegetative growth to reproductive growth [12]. Whether it can be carried out normally determines the number of flowers and seed quality of plants each year. Plants accurately combine internal signals with external environmental signals and then start the flowering process, which is particularly important for the whole process of plant growth and development [13]. In Arabidopsis, six important pathways were identified in the regulation of flower bud differentiation, including the photoperiod pathway, vernalization pathway, thermosensory pathway, autonomous pathway, gibberellin pathway, and age pathway [14]. These six regulation pathways are independent and cross-linked with each other, forming a precise regulatory network of flowering. The photoperiod is a very important condition that affects the growth and development of plants, and the entire life cycle of a plant is regulated by the photoperiod [15]. In the process of responding to the photoperiod, the *CONSTANS* (CO) and *FLOWERING LOCUS T* (*FT*) genes are important flowering promoting factors [16, 17]. At the early stage of vegetative growth, plants can speed up flowering under appropriate conditions after a period of low temperature treatment. This process of low temperature inducing plant flowering is called vernalization [18]. *FLOWERING LOCUS C* (*FLC*) is the key gene of the vernalization pathway, and the process of vernalization is the process of silencing the *FLC* [19]. In Arabidopsis, the autonomous pathway is independent of endogenous hormone regulation pathways, which promote flowering by inhibiting the expression of the *FLC* gene of the flower inhibitor [20]. Zhao proved that spraying high concentrations of GA has an obvious effect on the flowering of *M. micrantha* [21]. In addition to GA, other endogenous hormones, such as auxin, cytokinin, ethylene, abscisic acid, brassinosteroids, jasmonic acid and salicylic acid also play a positive or negative role in the signaling network leading to reproductive growth [22, 23].

The transcriptome refers to the sum of all RNAs transcribed by specific tissues and cells in a specific function or development period, including mRNAs and noncoding RNAs (ncRNAs) [24, 25]. Transcriptome technology has been successfully applied to the study of flower bud differentiation mechanisms in many plants, such as *Annona squamosa*, *Litsea cubeba*, and* Chrysanthemum morifolium* [26–28]. In the model plant *Arabidopsis thaliana*, the flowering mechanism has also been studied well and to extent in a few other plant species [29]. However, the information available about the molecular basis of flower bud differentiation in *M. micrantha* is very scarce. *M. micrantha* has different flowering numbers at different altitudes. Therefore, understanding the molecular mechanism of flower bud differentiation and mining genes related to flower bud differentiation in *M. micrantha* at different altitudes to formulate corresponding control measures are of great significance to the prevention and control of this invasive plant. In the later control process of *M. micrantha*, endophytes can be used to affect the expression of these excavated genes involved in regulating flower bud differentiation of *M. micrantha* to further affect its reproductive growth. In our study, we constructed independent cDNA libraries of three different altitudes in *M. micrantha* for Illumina RNA-seq. Analysis of transcriptome data related to bioinformatics was used to characterize flower bud transcriptional pathways at different altitudes of *M. micrantha*. We identified and mined the key differentially expressed genes (DEGs) that were subject to regulation in flower bud differentiation at different altitudes. Transcriptome sequencing of *M. micrantha* flower buds may help elucidate the role of various hormones, transcription factors, and regulatory pathways in flowering.

## Result

### Differences in the flowering number of *M. micrantha* at different altitudes

**Fig. 1:**
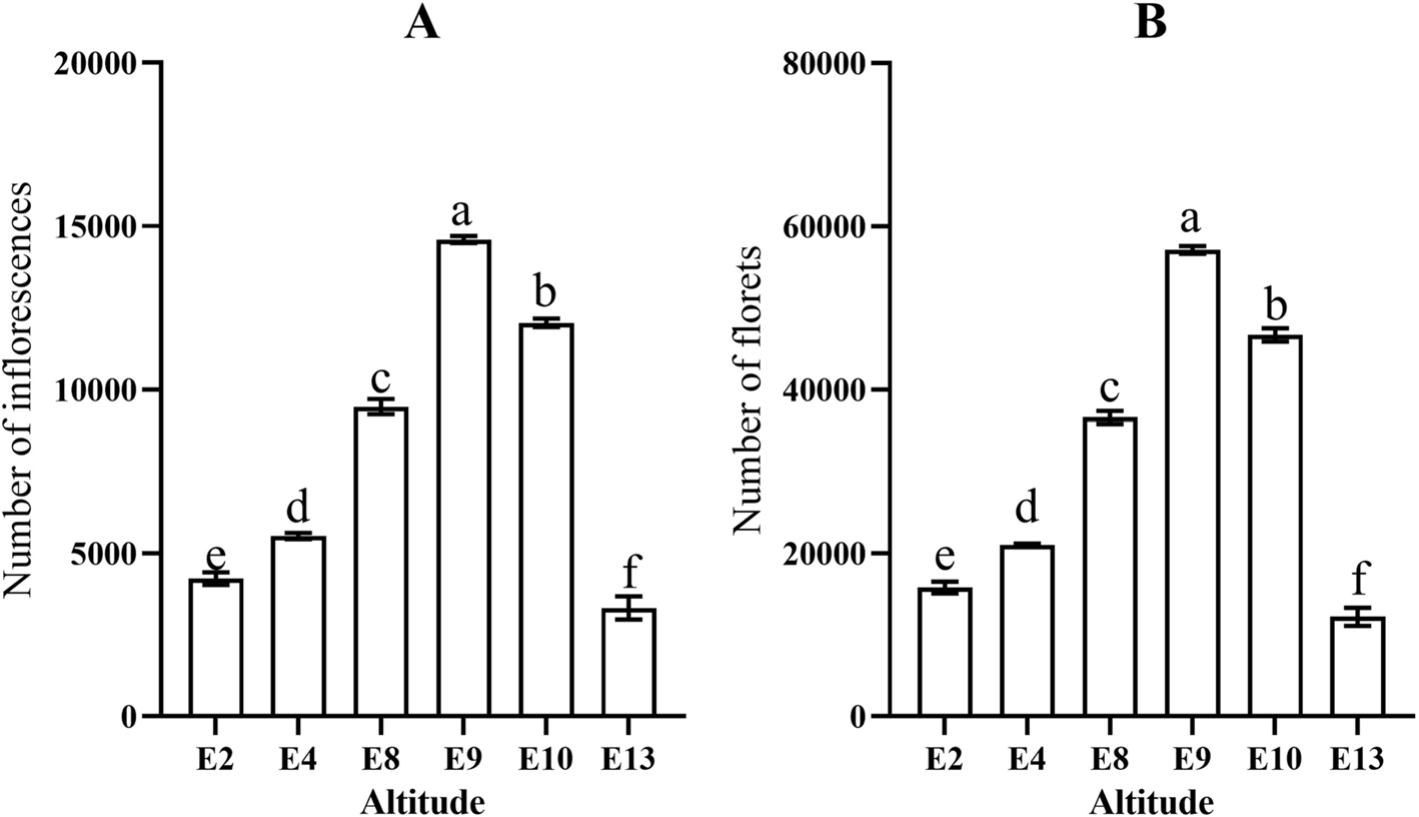
Differences in flowering number of *M. micrantha* at different altitudes. **A** The number of inflorescences of *M. micrantha* at different altitudes. **B** The number of florets of *M. micrantha* at different altitudes. Different lowercase letters indicate that there were significant differences in the number of inflorescences or florets of *M. micrantha* at different altitudes (*p* < 0.05)

AS shown in Fig. 1, there were differences in the flowering number of *M. micrantha* at different altitudes. We observed that the number of inflorescences and florets at 900 m was significantly higher than those at 200 m and 1300 m. The number of inflorescences at 900 m was five times that at 1300 m and 3.6 times that at 200 m. In addition, the number of inflorescences and florets at 200 m was greater than that at 1300 m, but the difference was not significant. Overall, in Dehong Dai and Jingpo Autonomous Prefecture, Yunnan Province, China, the most suitable growth area for *M. micrantha* is 900 m. High altitude and low altitude are not conducive to the flowering and invasion of *M. micrantha*. To further understand the molecular mechanism of flower bud differentiation in *M. micrantha*, we selected flower buds at the lowest altitude (200 m), the most suitable altitude (900 m) and the highest altitude (1300 m) for transcriptome analysis.

## Difference analysis of ecological factors related to altitude

**Fig. 2:**
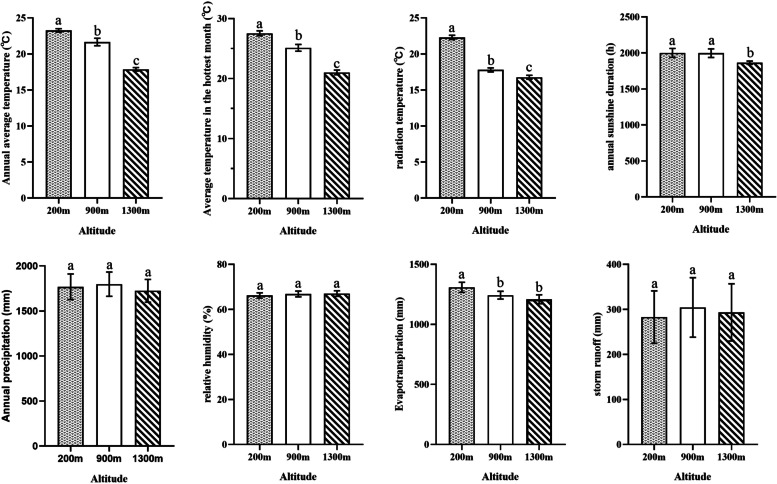
Bar chart of significance analysis of ecological factors at three altitudes. Different lowercase letters indicate significant differences in ecological factors at different altitudes (*p* < 0.05)

The significant differences in ecological factors in the three altitude areas invaded by *M. micrantha* were analyzed. The results showed (Fig. 2) that the annual average temperature, the average temperature of the hottest month and the radiation temperature in the high-altitude (1300 m) and low-altitude (200 m) areas invaded by *M. micrantha* were significantly lower than those at an altitude of 900 m. In high-altitude areas, the annual sunshine duration is significantly lower than 900 m. There was no significant difference in annual precipitation, relative humidity or storm runoff among the three altitude regions. Among the environmental factors related to altitude, temperature may be one of the most important factors affecting the ecological adaptability of *M. micrantha*. In addition to temperature, light may also significantly affect the flower bud differentiation of *M. micrantha*.

## Illumina sequence and assembly 

The transcriptome of *M. micrantha* flower buds at three altitudes (200 m, 900 m, 1300 m) was monitored using RNA-Seq. In total, nine cDNA libraries with three repetitions for each altitude were prepared and sequenced. A total of 51,479,370, 46,823,016 and 53,614,214 raw reads were generated for an altitude of 200 m, 46,985,372, 43,984,916 and 46,915,486 for an altitude of 900 m, and 50,071,842, 58,214,954 and 60,613,424 for an altitude of 1300 m. After removing adaptors, low quality and ambiguous sequences, the clean reads were all higher than 96.61%, and the Q20 values were all over 97.92%. The average Q30 and GC percentages were, 93.92% and 43.1%, respectively (Table 1).

**Table 1:** Statistical analysis of *Mikania micrantha* reads in nine libraries

Sample	Raw Read	Clean Read	Clean Base (Gb)	Q20 (%)	Q30 (%)	GC Content (%)	Reads Mapped
E2-1	51,479,370	50,184,502	7.53	97.99	93.78	43.10	89.05%
E2-2	46,823,016	45,537,886	6.83	98.14	94.14	43.10	88.84%
E2-3	53,614,214	52,370,142	7.86	98.11	94.07	43.12	88.64%
E9-1	46,985,372	45,730,652	6.86	97.99	93.81	43.08	85.77%
E9-2	43,984,916	42,891,540	6.43	98.03	93.91	43.10	83.92%
E9-3	46,915,486	45,908,178	6.89	98.05	93.96	43.02	86.99%
E13-1	50,071,842	48,757,468	7.31	97.92	93.66	42.68	77.35%
E13-2	58,214,954	56,240,314	8.44	98.02	93.97	43.55	78.16%
E13-3	60,613,424	59,078,680	8.86	98.06	94.00	43.15	72.54%

Q20 is the proportion of nucleotides with a quality value > 20; Q30 is the proportion of nucleotides with a quality value > 30; GC is the proportion of guanidine and cytosine nucleotides present

**Fig. 3:**
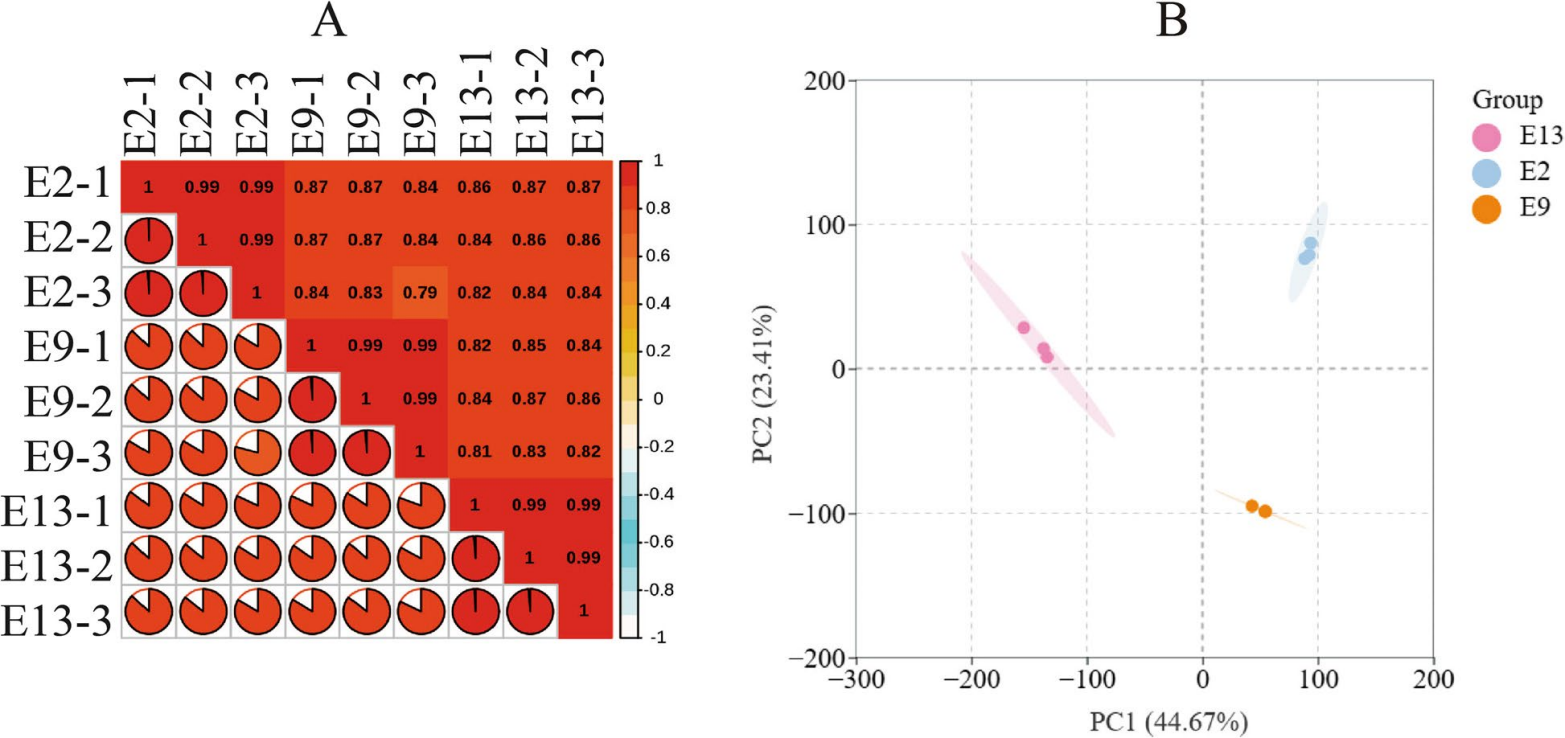
Reliability analysis of biologically repeated samples. **A** Pearson correlation coefficients of all nine samples. The Pearson correlation coefficients of all gene expression levels at E2, E9, and E13; **B** Principal component analysis of expressed genes in floral bud differentiation of M. micrantha at different altitudes, i.e., E2, E9, and E13

The Pearson correlation coefficients of all gene expression values between each pair of samples were calculated, and the correlation coefficients of the nine datasets are presented in the form of heatmap (Fig. 3A). These graphs reflect the correlations of gene expression at three altitudes, the higher the correlation coefficient is, the more similar the gene expression level is. Principal component analysis (PCA) showed that there were significant differences in gene expression profiles among the treatment repeat groups at the three altitudes. PC1 and PC2 explained 44.67% and 23.41% of the variation, respectively (Fig. 3B).

## Differential gene expression analysis

**Fig. 4:**
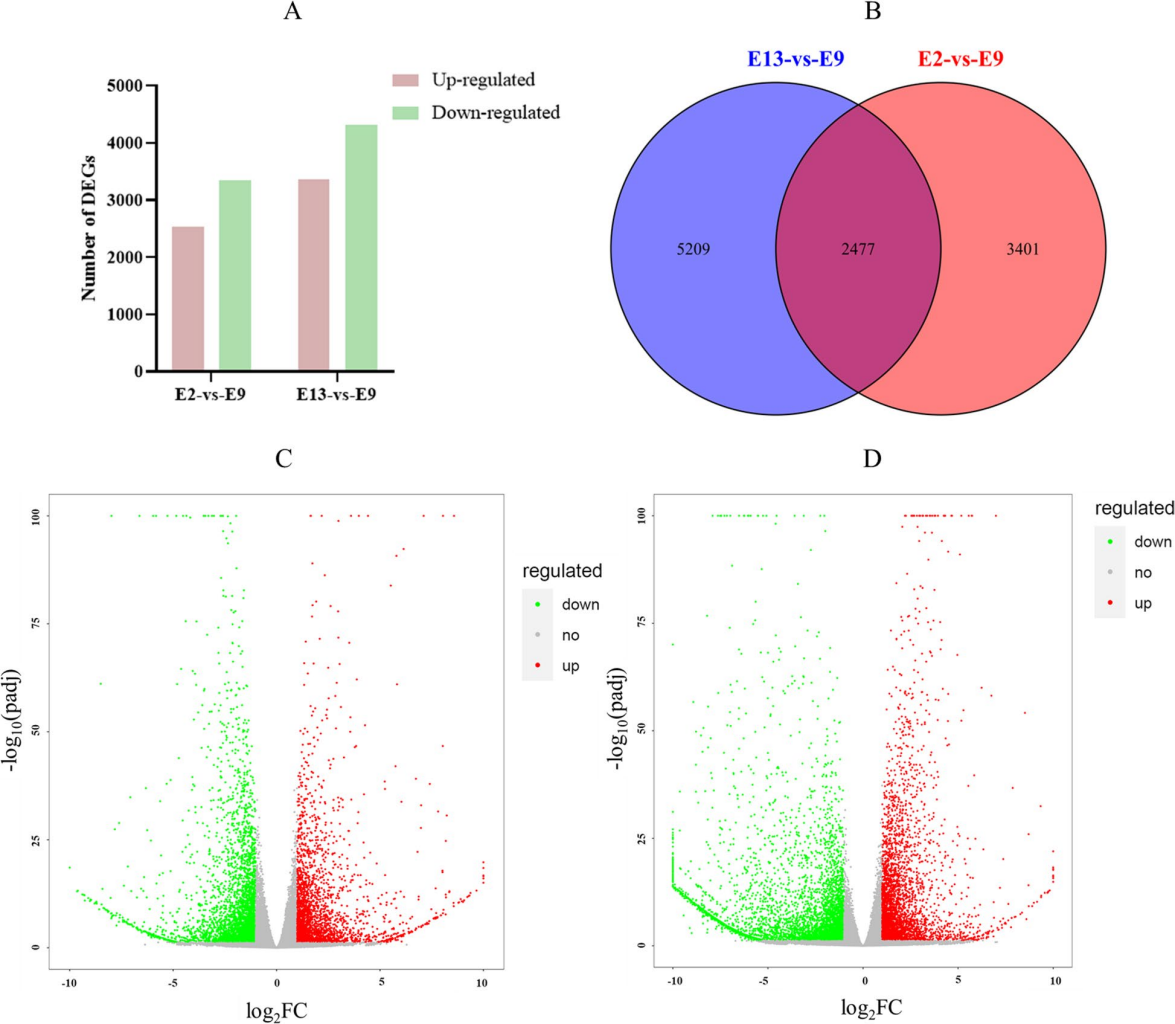
Analysis of the DEGs at different altitudes in *M. micrantha*. **A** Venn diagram of DEGs included in two comparisons. **B** DEGs identified from different comparisons. **C** Volcano map of E2vs-E9. **D** Volcano map of E13- vs-E9

In the transcriptomes obtained at different altitudes of *M. micrantha*, we set the following criteria for DEG screening: |log2Fold Change|≥ 1 and Q value ≤ 0.05. As shown in Fig. 4, in the E2 vs. E9 comparison, 5878 differentially expressed; 2527 upregulated and 3351 downregulated unigenes were found. For the E13 vs. E9 comparison, there were 7686 differentially expressed gene, of which 3365 were upregulated and 4321 downregulated (Fig. 4A). Meanwhile, the Venn diagram of the DEGs shows the overlap relationships between the comparison groups, 2477 DEGs were identified in both E2 vs. E9 and E13 vs. E2 (Fig. 4B). In the volcano plot, red dots are upregulated DEGs, green dots are downregulated DEGs, and the number of downregulated DEGs was greater than the number of upregulated DEGs in E2 vs. E9 and E13 vs. E9 (Fig. 4C and D). Moreover, we screened out some of the most highly expressed genes from the volcano map by the following rules: -8 ≥ log_2_Fold change or log_2_Fold change ≥ 8 and then analyzed and described these genes by KEGG annotation. After KEGG annotation of these most highly expressed genes, it was found that a large number of genes were enriched in metabolic pathways and biosynthesis of secondary metabolites (Additional file 1).

## GO and KEGG enrichment analyses of DEGs

To study the *M. micrantha* bud transcriptome dynamics and identify the candidate genes, three transcriptomes were generated from different altitude samples (200 m, 900 m, and 1300 m). In this work, GO and KEGG analyses were used to classify the functions of the annotated DEGs under different altitude treatments.

**Fig. 5:**
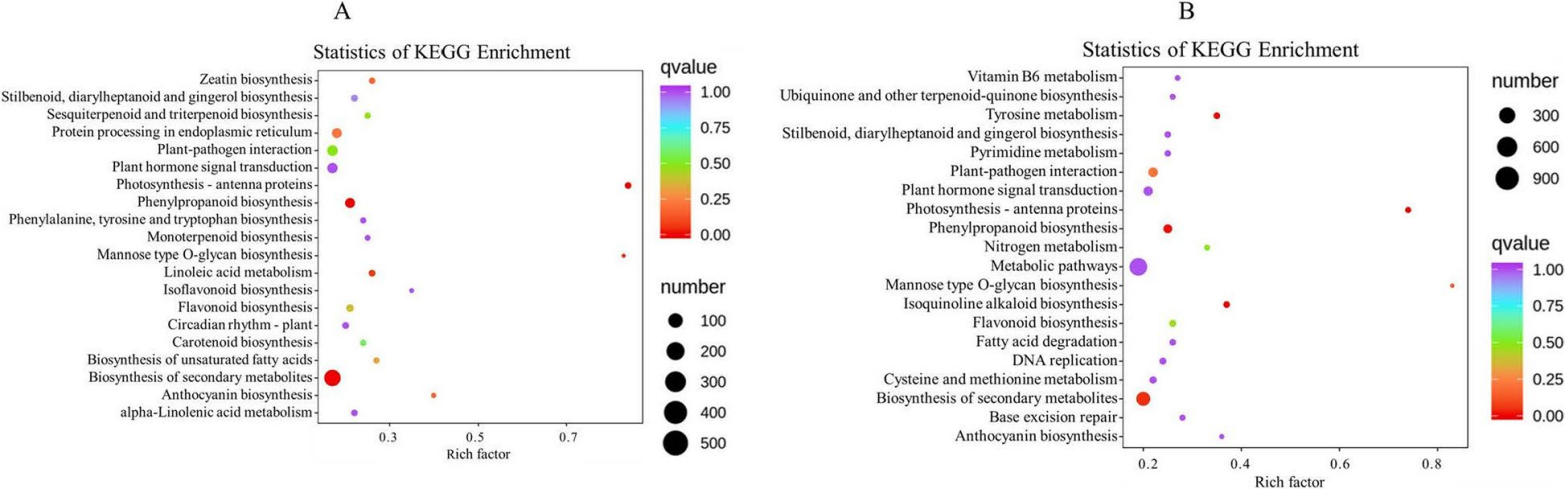
KEGG Enrichment Analyses of DEGs. **A** E9 vs. E2 Kyoto Encyclopedia of Genes and Genomes (KEGG) pathway enrichment analyses of DEGs. **B** E9 vs. E13 Kyoto Encyclopedia of Genes and Genomes (KEGG) pathway enrichment analyses of DEGs. Rich factor is a ratio of the number of DEGs annotated with a pathway relative to the total number of genes annotated with this pathway. The larger the value of the rich factor, the greater the enrichment of this KEGG pathway

GO and KEGG pathway enrichment analyses were performed on the DEGs to identify differences in the biological processes and pathways between E2 and E9. In total, many DEGs between E9 and E2 were enriched in GO categories. As shown in Additional file 2, GO enrichment analysis of the biological processes category identified a number of categories, including 'secondary metabolite biosynthetic' (GO:0,044,550), 'hormone biosynthetic process' (GO:0,042,446) and 'host programmed cell death induced by symbiont' (GO:0,034,050). For the 'molecular function' category, the dominant terms were 'ADP binding' (GO:00,435,310), 'oxidoreductase activity, acting on CH-OH group of donors' (GO:0,016,614), and 'glucosyltransferase activity' (GO:0,046,527). For the 'cellular component' category, the dominant term was 'chromatin'(GO:0,000,785) Furthermore, 2064 DEGs enriched for KEGG pathways between E2 and E9 (Additional file 3), 'plant hormone signal transduction' (ko04075), 'biosynthesis of secondary metabolites' (ko01110), 'phenylpropanoid biosynthesis' (ko00940), and 'plant-pathogen interaction' (ko04626) were the main different pathways between these two samples (Fig. 5A). The hormones and some secondary metabolites provide material and energy for flower bud differentiation, and the enrichment results indicated that the genes involved in plant hormone signal transduction and secondary metabolism were different at different altitudes, which suggested that the materials and energy involved in bud differentiation of *M. micrantha* were different at different altitudes.

Additionally, many DEGs were enriched in GO categories between E13 and E9. In the 'biological process' category, the dominant terms were the following: 'anion transport' (GO:0,006,820), 'hormone metabolic process' (GO:0,042,445), and 'response to cold' (GO:0,009,409). In the 'cellular component' category, the most representative term was the following: 'plant-type cell wall' (GO:0,009,505). Finally, in the 'molecular function' category, the most representative terms were the following: 'oxidoreductase activity, acting on CH-OH group of donors' (GO:0,016,614), 'ADP binding' (GO:0,043,531), and 'hydrolase activity, hydrolyzing O-glycosyl compounds' (GO:0,004,553) (Additional file 2). In addition, 2682 DEGs enriched for KEGG pathways between E13 and E9 (Additional file 4), 'metabolic pathways' (ko01100), 'biosynthesis of secondary metabolites' (ko01110), 'plant hormone signal transduction' (ko04075), 'phenylpropanoid biosynthesis' (ko00940), and 'plant-pathogen interaction' (ko04626) were the main enrichment pathways between these two samples (Fig. 5B).

## Identification of DEGs involved in flowering and various flowering-related hormones pathways

**Fig. 6:**
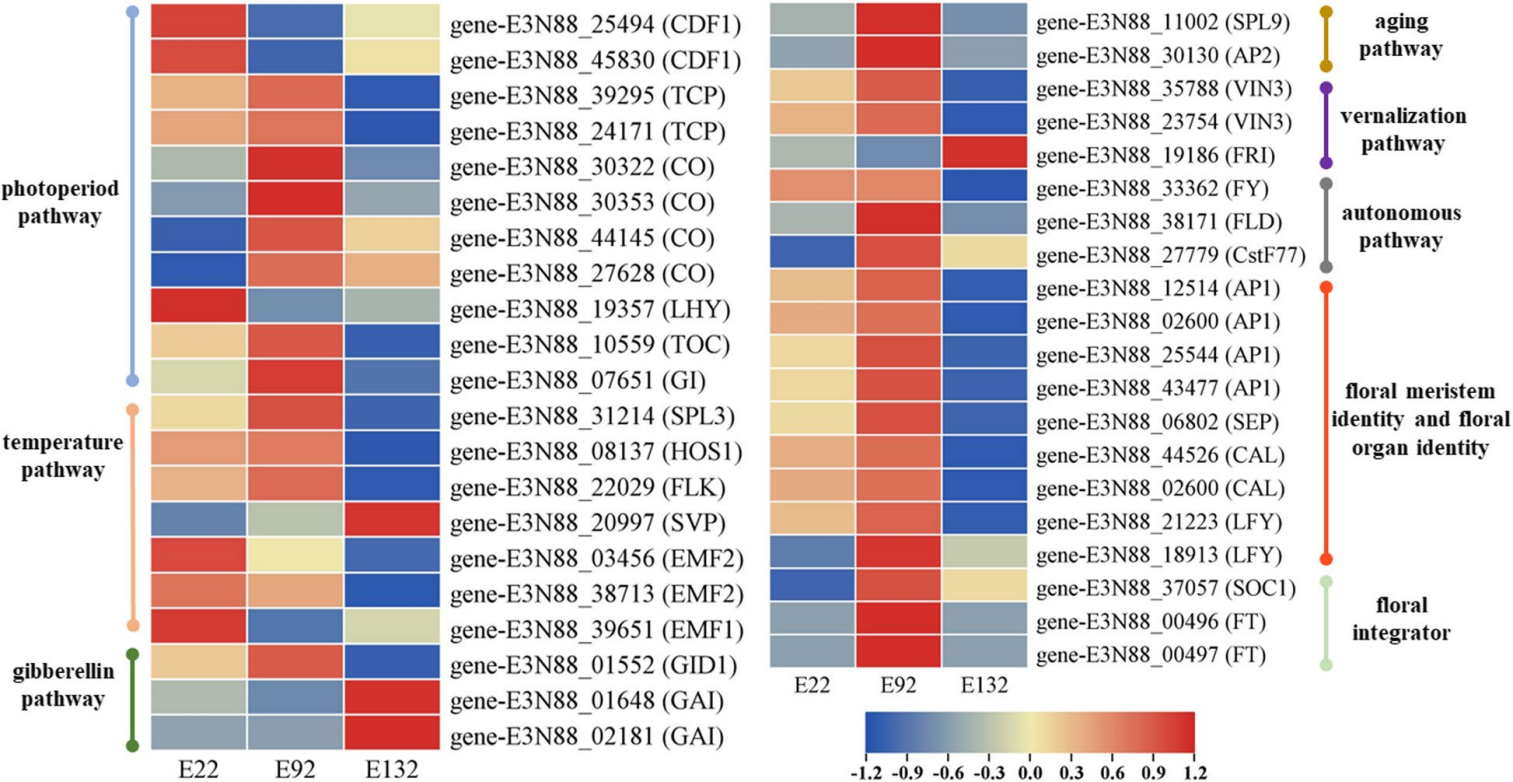
Expression changes of the DEGs involved in flowering-related pathways

We further analyzed the expression levels of key DEGs of the flowering pathway and flower molecular networks in flower development. Based on a comparative analysis of the NCBI and TAIR databases, many DEGs in *M. micrantha* showed homology to known flowering time-associated genes from other plant species. Most of the flowering time associated genes in *M. micrantha* could be assigned to six classical flowering-related pathways (Fig. 6 and Additional file 5).

In the photoperiod pathway, we identified nine DEGs, *GIGANTEA* (*GI*, 1 DEG), *CYXLIC DOF FACTOR 1* (*CDF1*, 2 DEGs), *CONSTANS* (*CO*, 4 DEGs), and *TEOSINTE BRANCHED 1/CYCLOIDEA/PCF* (*TCP*, 2 DEGs), which were significantly upregulated at 900 m except for *CDF1*. Interestingly, we identified two unigenes that affect the circadian rhythm of the circadian clock, which included *LATE ELONGATED HYPOCOTYL* (*LHY*) and *TIMING OF CAB OF EXPRESSION 1* (*TOC1*). *LHY* downregulated at 900 m. Additionally, in the gibberellin pathway, we identified four key genes, including *GIBBERELLIN RECEPTOR GID1* (*GID1*, 2 unigenes) and *GA RECEPTOR DELLA PROTEIN* GIBBERELLIC ACID INSENSITIVE1 (GAI, 2 DEGs). Among them, *GID1* was significantly upregulated at 900 m, and two GAIs were downregulated at 900 m. Additionally, three DEGs in *M. micrantha* were involved in the autonomous pathway, *FLOWERING TIME CONTROL PROTEIN FY* (*FY*, 1 DEG), *FLOW-ERING LOCUS D* (*FLD*, 1 DEG), and *CLEAVAGE STIMULATION FACTOR SUBUNIT 77* (*CstF77*, 1 DEG), which were upregulated at 900 m. In addition, several *M. micrantha* DEGs exhibited similarities to genes in the vernalization pathway, including *FRIGIDA* (*FRI*, 1 DEG) and *VERNALIZATION IN-SENSITIVE 3* (*VIN3*, 2 DEGs). For the age pathway, only two DEGs were found, including *SQUAMOSA PROMOTER-BINDING-LIKE PROTEIN 9* (*SPL9*, 1 DEG) and *APETELA 2* (*AP2*, 1 DEG), which were evidently upregulated at 900 m. Finally, we identified key genes in the temperature pathway including *SQUA-MOSA PROMOTER-BINDING-LIKE PROTEIN 3* (*SPL3*), *HIGH EXPRESSION OF OS-MOTICALLY RESPONSIVE GENES 1* (*HOS1*), *FLOWERING LOCUS K* (*FLK*), and *EMBRYONIC FLOWER 1* (*EMF1*). However, none of them were DEGs. Among them they were upregulated at 900 m except for *SVP*. In addition to the genes related to the six pathways, we obtained some DEGs associated with flower integrator genes *FLOWERING LOCUS T* (*FT*) and *SUPPRESSOR OF CONSTANS OVEREXPRESSION 1* (*SOC1)*, flower meristem identity genes *LEAFY* (*LFY*), flower organ identity genes *CAULIFLOWER* (*CAL*), *APETALA 1* (*AP1*), and *SEPALLATA* (*SEP*) that were identified and were found to be mainly upregulated at 900 m.

**Fig. 7:**
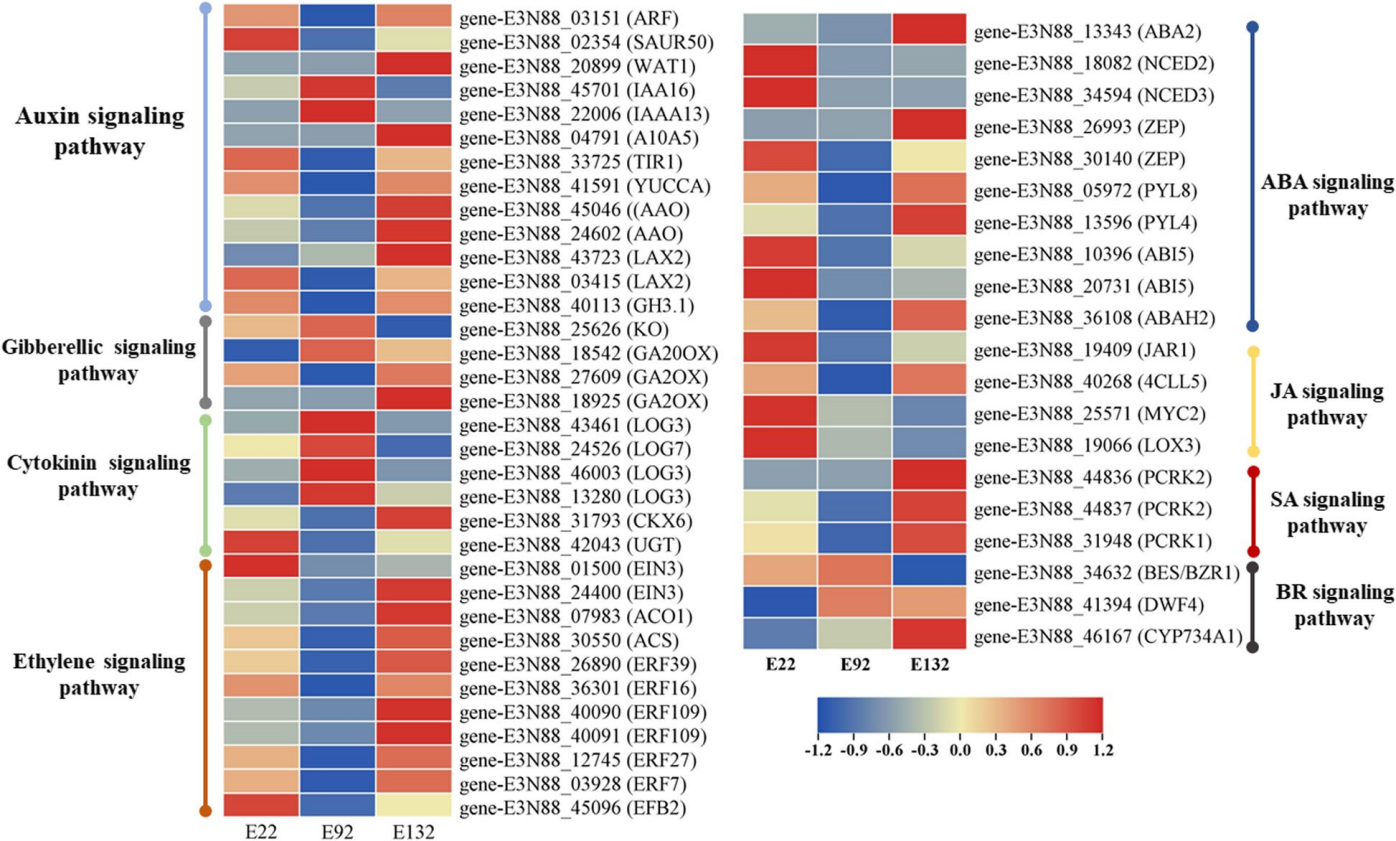
Expression levels of DEGs involved in the auxin signaling, brassinosteroid signaling, gibberellic signaling, cytokinin signaling, ABA signaling, JA signaling, SA signaling, and ethylene signaling pathways

Transcriptional levels of hormone signal transduction showed the DEGs involved in the signal transduction pathways of auxin, GA, cytokinin (CK), ethylene, ABA, BR, JA, and SA at three elevations in *M. micrantha *(Fig. 7 and Additional file 6). At three elevations, we identified a total of 13 key DEGs in the auxin-signaling pathway, of which ten were significantly downregulated at 900 m. In contrast, two DEGs, i.e., IAA16 and IAA13, were upregulated at 900 m. In the brassinosteroid biosynthetic process, we identified three key genes, including *CYTOCHROME P450 734A1* (*CYP734A1*), *Dwarf 4* (*DWF4*), and *BRASSI-NAZOLE-RESISTANT 1* (*BZR1*). Interestingly, they were upregulated at 900 m. We also identified four key genes in gibberellin biosynthetic processes and metabolism, of which *GIBBERELLIN 20-OXIDASE* (*GA20OX*) and *ENT-KAURENE OXIDASE* (*KO*) were upregulated at 900 m, while *GIBBEREL-LIN-2-BETA-DIOXYGENASE* (*GA2OX*) were downregulated at 900 m. Additionally, compared with E9, we identified many key genes in the ethylene signaling pathway, of which *ETHYLENE INSENSITIVE 3* (*EIN3*), *1-AMINOCYCLOPROPANE-1-CARBOXYLATE OXIDASE HOMOLOG 1* (*ACO1*), *1-AMINOCYCLOPRAPANE-1-CARBOXYLATE SYNTHASE* (*ACS*), *ETH-YLENE-RESPONSIVE TRANSCRIPTION FACTOR 039* (*ERF39*), *ETHYLENE RESPON-SIVE TRANSCRIPTION FACTOR 016* (*ERF16*), *ETHYLENE RESPONSIVE TRAN-SCRIPTION FACTOR 109* (*ERF109*), *ETHYLENE RESPONSIVE TRANSCRIPTION FACTOR 027* (*ERF27*), and *ETHYLENE RESPONSIVE TRANSCRIPTION FACTOR 07* (*ERF7*) were significantly upregulated at 1300 m. In cytokinin signaling pathway, six key genes were identified, of which *LONELY GUY 7* (*LOG7*) and *LONELY GUY 3* (*LOG3*) were upregulated at 900 m, of which *CYTOKININ DEHYDROGENASE 6* (*CKX6*) and UDP-*GLYCOSYLTRANSFERASE* (*UGT*) were downregulated at 900 m. In the salicylic acid signaling, we found that *PTI-COMPROMISED RECEPTOR-LIKE CYTOPLASMIC KI-NASE 1* (*PCRK1*), and *PTI-COMPROMISED RECEPTOR-LIKE CYTOPLASMIC KI-NASE 2* (*PCRK2*), which were significantly downregulated at the 900 m. One *JASMONIC ACID-AMIDO SYNTHETASE* (*JAR1*) gene, *4-COUMARATE-COA LIGASE-LIKE 5* (*4CLL5*) gene, LIPOXYGENASE 3 (*LOX3*), and *MYC2* were downregulated at 900 m in the jasmonic acid (JA) signaling pathway. In the abscisic acid biosynthetic process, ten key genes i.e., *XaNTHOXIN DEHYDROGENASE* (*ABA2*), *9-CIS-EPOXYCAROTENOID DIOXYGENASE 3* (*NCED3*), *9-CIS-EPOXYCAROTENOID DIOXYGENASE 2* (*NCED2*), *ZEAXANTHIN EPOXIDASE* (*ZEP*), *ABSCISIC ACID RECEPTOR PYL8, ABSCISIC ACID RECEPTOR PYL4, ABSCISIC ACID-INSENSITIVE 5* (*ABI5*), and *ABSCISIC ACID 8'-HYDROXYLASE 2* (*ABAH2*) were downregulated at 900 m.

## Measurements of endogenous hormone concentrations

**Fig. 8:**
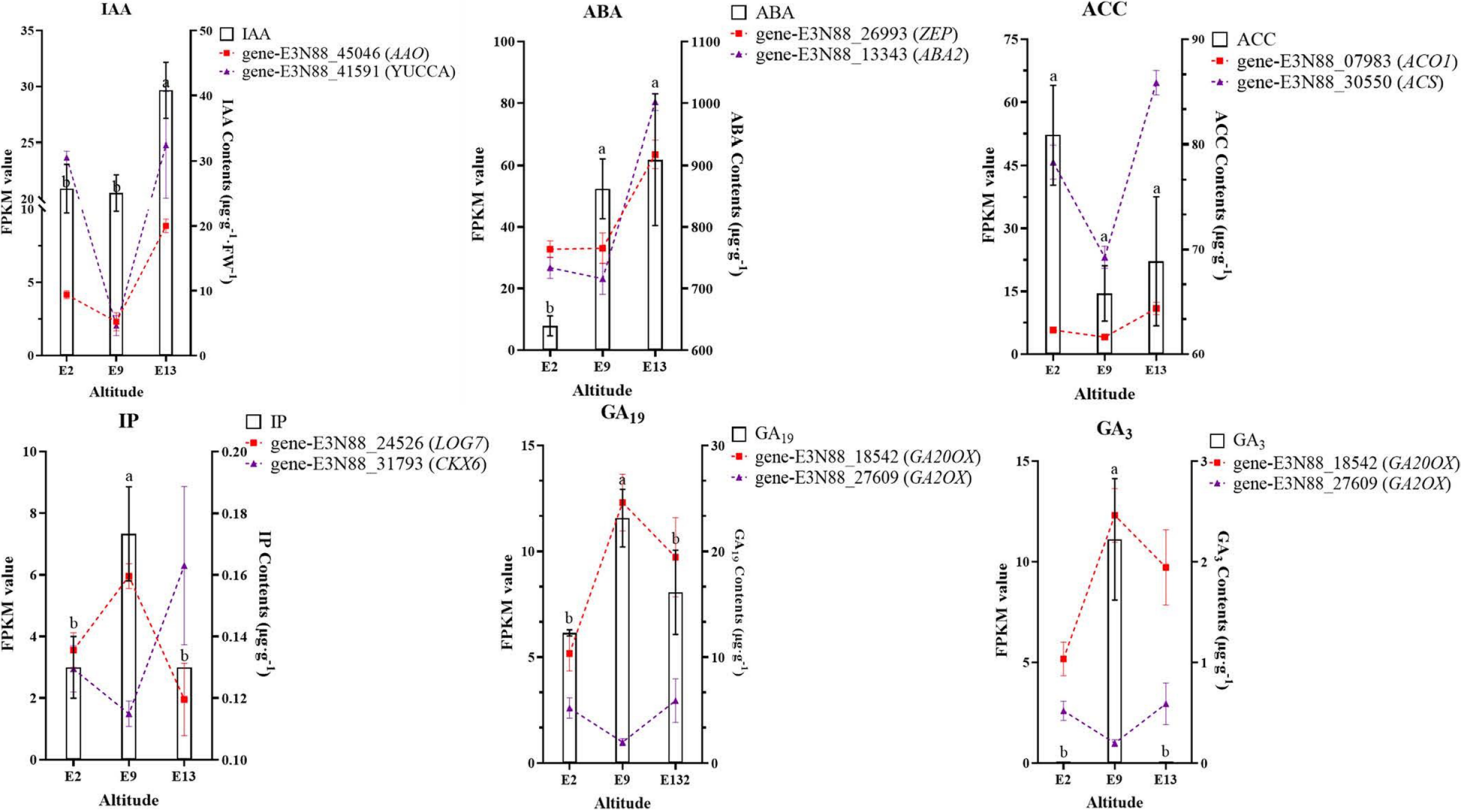
Changes in endogenous hormone content at different altitudes. **A** ABA contents, **B** ACC contents, **C** IAA contents, **D** GA19 contents, **E** GA3 contents, and **F** IP contents at three different altitudes in M. micrantha were measured. Significant differences are indicated by different letters (*p* < 0.05)

There are many kinds of plant hormones in flower buds, and different plant hormones play different regulatory roles in the process of plant flowering. The results of the KEGG annotation urged us to measure the levels of several plant endogenous hormones in the flower buds of *M. micrantha* at different altitudes To examine the roles of endogenous hormones at flower bud differentiation in *M. micrantha*, the contents of abscisic acid (ABA), 1-aminocyclopropanecarboxylic acid (ACC), gibberellins GA_S_, indole acetic acid (IAA), jasmonic acid (JA), salicylic acid (SA), brassinosteroids (BR), trans-zeatin (tZ), cis-zeatin (cZ), and N6-isopentenyladenine (IP) were measured in *M. micrantha* flower buds at different elevations. The content of JA, SA, BR, tZ, and cZ were too low to be analyzed. Both the GA_19_, GA_3_, and IP contents reached their highest points at 900 m. It was also found that the changes in the contents of the selected DEG (gene-E3N88_27609) involved in GA_19_ and GA_3_ metabolism at different altitudes were opposite to the changes in GA_19_ and GA_3_ contents at different altitudes. However, the ACC and IAA contents reached their lowest point at 900 m. The IAA content in the E13 area was significantly higher than that in the E9 area by 62.74%, and the ACC content in the E13 area was higher than that in the E9 area by 4.67%. It was also found that the content changes of the selected two DEGs (gene-E3N88_26993 and gene-E3N88_13343) involved in IAA synthesis and two DEGs (gene-E3N88_07983 and gene-E3N88_30550) involved in ACC synthesis at different altitudes were consistent with the change trend of IAA content at different altitudes. The content of ABA increased significantly from 639.33 μg·g^−1^ (200 m) to 101.32 μg·g^−1^ (1300 m), which reached their highest point at 1300 m (Fig. 8). Both the ABA content and the expression of DEGs (gene-E3N88_26993 and gene-E3N88_13343) involved in ABA synthesis were the lowest in E9. This indicated that various endogenous hormones are closely related to flower bud differentiation of *M. micrantha*.

## Bioregulation analysis and WGCNA of DEGs

**Fig. 9:**
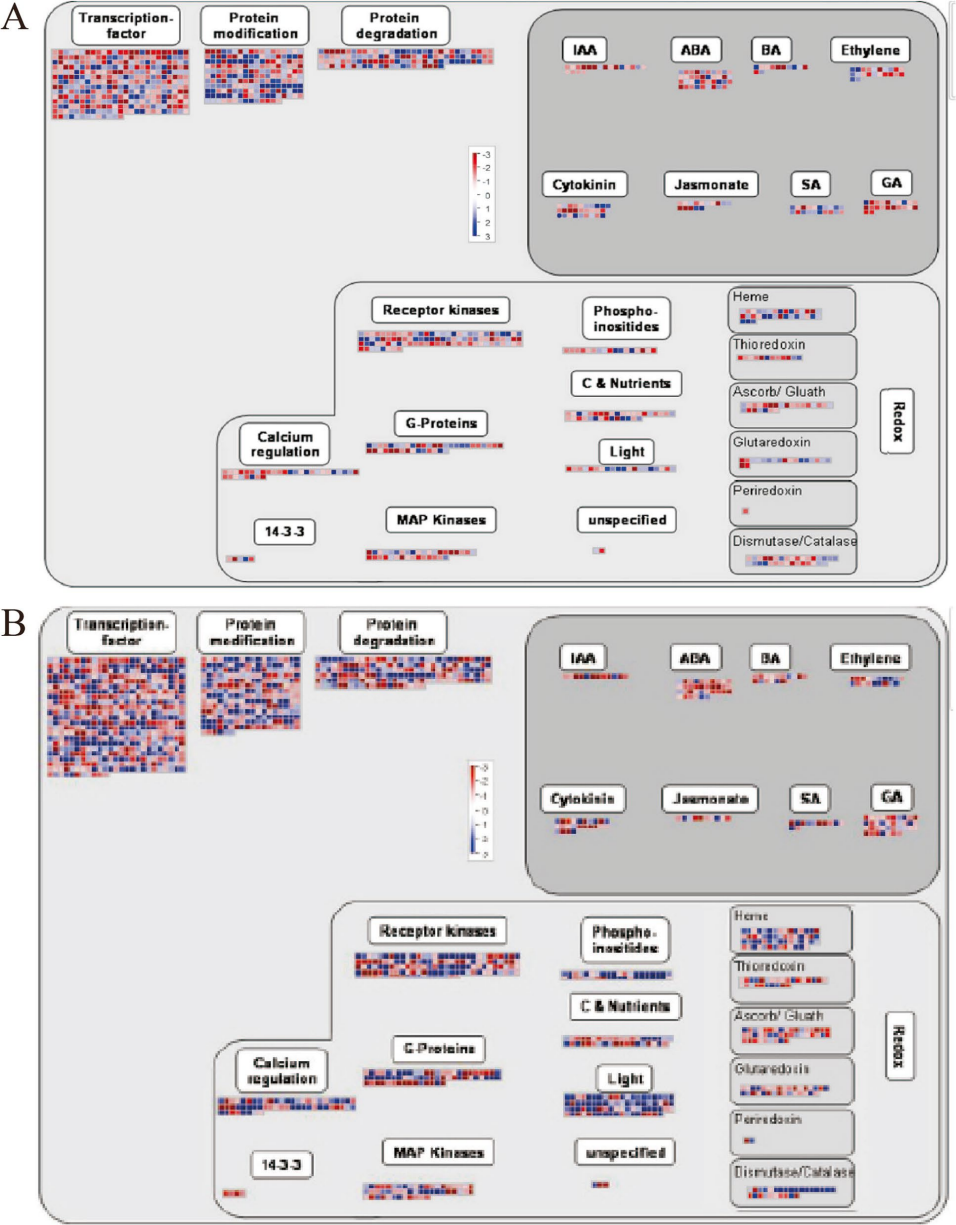
The clustering pattern of DEGs generated with the MapMan tool. **A** Overview of DEG regulation in E9-vs-E2. **B** Overview of DEG regulation in E9-vs-E13. Each square represents a separate gene. Red represents upregulation, and blue represents downregulation. The color brightness represents the degree of difference

Using MapMan software, the biological regulation and metabolism-related roles of the DEGs were analyzed at different altitudes of *M. micrantha* flower buds (Fig. 9). The results showed that the DEGs mainly responded to IAA, ethylene, ABA, cytokinins, BR, JA, SA, and GA hormone regulation. There were many upregulated and downregulated DEGs associated with various endogenous hormones, and there were more upregulated DEGs than downregulated DEGs. The DEGs involved in the ABA pathway and GA pathway were the most abundant, and the DEGs involved in the jasmonate pathway were the least abundant. However, many DEGs were enriched in the transcription factor (TF), protein modification, and protein degradation categories. TFs are key regulatory proteins of transcription in biological processes, especially in flower bud differentiation. Therefore, we studied the expression dynamics of TF genes in *M. micrantha*. In total, 444 TF genes were identified in E9 vs. E2 and 592 TF genes were identified in E9 vs. E13. In this study, the top ten most abundant genes during flower bud differentiation included basic helix-loop-helix (bHLH), NAC, B3, MYB, basic leu-cine zipper (bZIP), WRKY, ERF, MADS-box, C2H2, and MYB-related genes. For redox reactions, the DEGs participated in biological reactions regulated by calcium regulation, receptor kinases, G-proteins, MAP kinases, phosphoinositides, carbon, nutrients, and light. Several DEGs accumulated in a series of oxidoreductase categories, such as the heme, thioredoxin, ascorbate/glutathione, glutaredoxin, and dismutase/catalase categories. In addition, the DEGs participated in biological reactions regulated by light, nutrients, carbon, phosphoinositides, MAP kinases, G-proteins, receptor kinases, and calcium regulation, and 14.3.3 participated in redox reactions.

**Fig. 10:**
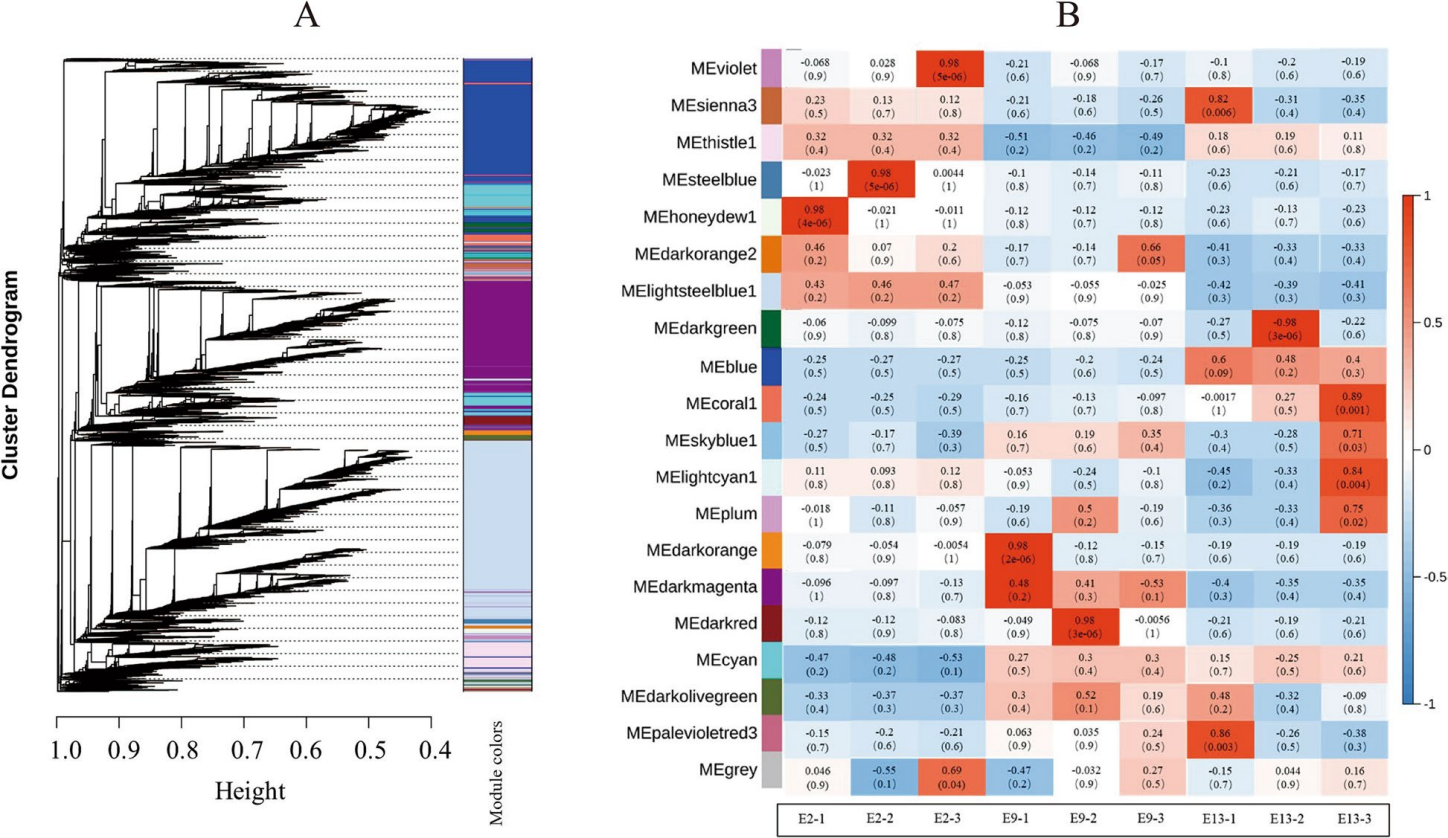
Gene dendrogram module colors and module–trait relationships identified by weighted gene coexpression network analysis (WGCNA) in *M. micrantha* flower bud samples. **A** Gene clustering tree and module division. In the dendrogram, each leaf corresponds to a gene. **B** Module-trait associations. The abscissa represents the samples; the ordinate represents the modules. The upper value in each cell represents the correlation coefficient, and the lower value represents the *p* value

Weighted correlation network analysis (WGCNA) is an analytical method to explore the relationship between modules and concerned phenotypes and to mine key genes in coexpression networks [30]. Many different modules were identified based on pairwise correlations of gene expression across the three samples. Eigengenes and clusters were calculated based on the correlations to quantify the coexpression similarity of entire modules, using a strict cutoff of 0.25, corresponding to a correlation of 0.75, to merge the optimization into 20 modules (Fig. 10).

Observing the correlation coefficients of the modules, it was found that the genes in the darkmagenta, and thistle had higher specificity at E9. The expression trend of differentially expressed genes on dark magenta module was upregulated at E9 and downregulated at E2 and E13. KEGG pathway analysis was performed in the dark magenta module, and it was found that DEGs were enriched in 'metabolic pathways', 'biosynthesis of secondary metabolites', 'starch and sucrose metabolism', and 'plant hormone signal transduction' (Additional file 7). This indicated that the flower bud differentiation of *M. micrantha* requires carbohydrate metabolism to provide energy. In addition, plant hormones also play a very important role in the flower bud differentiation of *M. micrantha*. In the starch and sucrose metabolism pathway (map00500), we found that many genes were highly expressed at E9, such as beta glucosidase (gene-E3N88_07917, gene-E3N88_09007, and gene-E3N88_09041) involved in D-Glucose synthesis, beta beta-fructofuranosidase (gene-E3N88_12860, gene-E3N88_27263, gene-E3N88_31592, gene-E3N88_33447, gene-E3N88_37860) involved in D-fructose synthesis, and sucrose synthase (gene-E3N88_25059) involved in sucrose synthesis, which were upregulated at E9. This suggests that these materials may provide the carbon source and energy for the flower bud differentiation of *M. micrantha* at E9.

## Discussion

### Illumina sequencing and sequence annotation

We performed comparative analysis of gene expression at three altitudes. We identified a number of DEGs involved in various biological processes, which are likely to be associated with the regulation of *M. micrantha* flower bud development. The number of downregulated genes was larger than upregulated genes in E2 vs E9 and E13 vs E9, indicating that flower bud differentiation requires more gene suppression than activation in *M. micrantha*. The onset of flower development was accompanied by the repression of many genes similar to those previously reported in Arabidopsis during the initiation of flower bud differentiation.

The second-generation molecular sequencing technology RNA-seq has already become the most common method used for studying gene expression and screening and predicting candidate genes [31]. Wang et al. (2021) used transcriptome sequencing to obtain candidate genes related to early flowering and flower development in two flower development stages of two tomato varieties with different flowering periods [31]. Zhang et al. (2013) screened genes with high differential expression and related flower development in *Cymbidium sinense* from three sublibraries as candidate genes [32]. Temporal regulation of gene expression plays an important role in plant growth and development.

## Identification of DEGs associated with flower bud differentiation and flower development

**Fig. 11:**
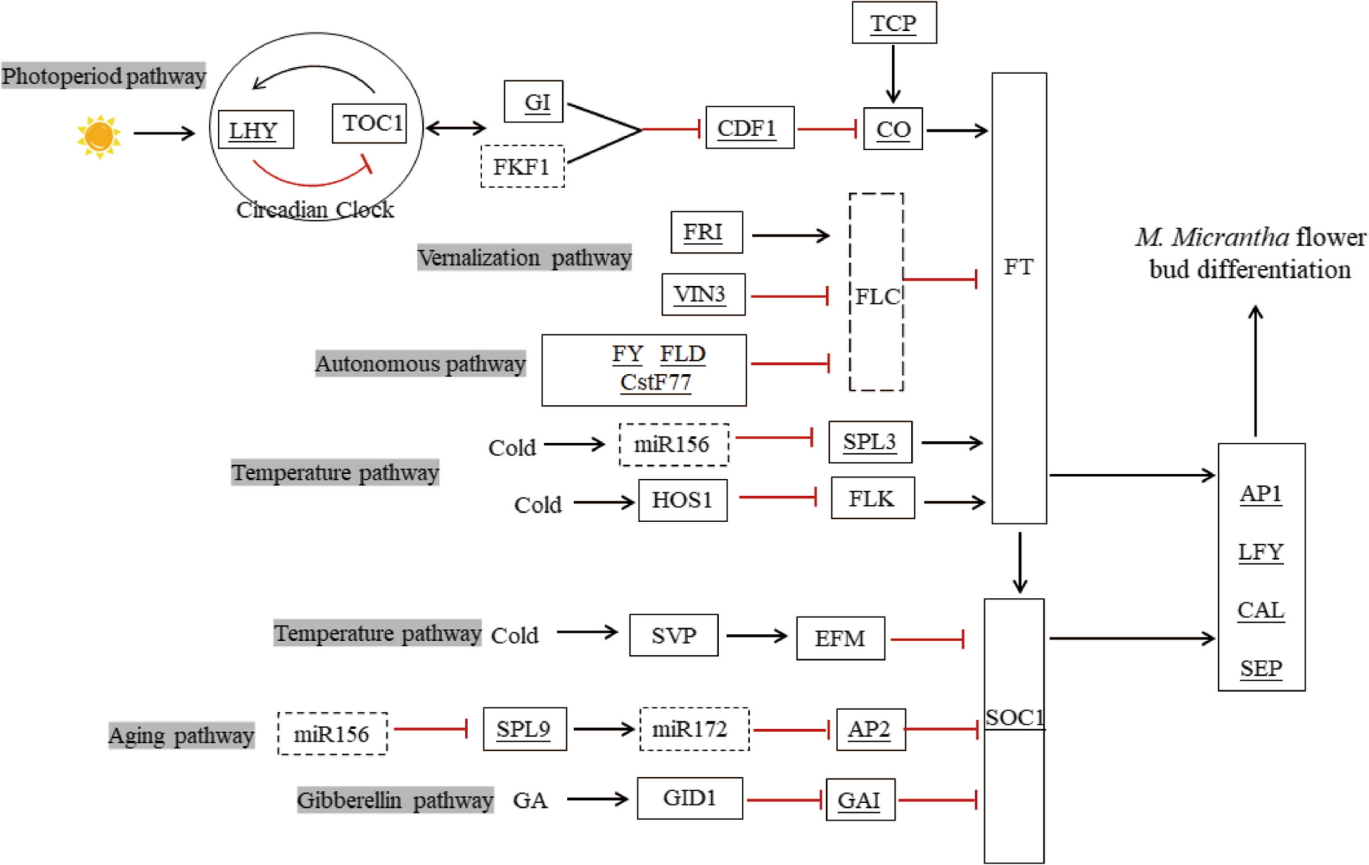
Schematic of the regulatory genes and pathways involved in floral bud differentiation of M. micrantha. The letters in the boxes represent gene abbreviations. The gene name with underlines indicates DEGs. The dashed box indicates that this gene was not found in our transcriptome. Lines with arrows indicate activation, and red lines with blunt ends indicate repression

Based on the regulatory network of flowering pathway related genes involved in Arabidopsis flower bud differentiation, six regulatory pathways were implicated in the response of *M. micrantha* (Fig. 11).

The photoperiod pathway includes the input pathway of the clock, a circadian clock and an output pathway from the clock to flowering [33]. Under long conditions, the expression of *CO* is modified by the activity of three proteins: *GI*, *FKF1*, and *CDF1*, which are regulated by circadian clock [34, 35]. In *M. micrantha*, *GI* is regulated by the circadian rhythm and activates the upregulated expression of downstream *CO* and *FT* by inhibiting the expression of *CDF1* at E9. The autonomic pathway is the flowering pathway by which some plants will bloom after reaching a certain stage of vegetative growth in the absence of external induction [20]. Seven regulators (*FCA*, *FY*, *FPA*, *FVE*, *FLD*, *FLK*, and *LD*) in the autonomous pathway of Arabidopsis inhibit the expression of *FLC* in a parallel manner to promote flowering [36, 37]. In *M. micrantha*, we only identified that *FY* and *FLD* were significantly upregulated at 900 m. Therefore, in the autonomous pathway of *M. micrantha*, the flower bud differentiation occurring at 900 m may be due to the inhibitory effect of the autonomous factors (*FY* and *FLD*) on *FLC*. In Arabidopsis, *FRI* is located upstream of *FLC* and mediates vernalization by regulating *FLC* [38, 39]. In our study, we found that *FRI* was upregulated at 1300 m, which may result in high expression of *FLC* at 1300 m. However, we did not detect *FLC* expression at any elevation, which may be due to sampling time. *Chenopodium Quinoa* Willd also did not identify a *FLC* homologous gene [40]. This result may implied that the expression of *FRI* is positively regulated to inhibit flower bud differentiation of *M. micrantha* at 1300 m. In biennials and winter annuals, *VIN3*, *VRN1*, and *VRN2* participate in the maintenance of *FLC* inhibition [41, 42]. We only found that a *VIN3* downregulated at 1300 m, Which may can promoted *FT* gene expression by inhibiting the *FLC* gene in *M. micrantha*. This result may implied that *VIN3* is a positive gene for flower bud differentiation of *M. micrantha*. In *Raphanus sativus*, genes related to the age pathway were also found, such as *SPL3*, *SPL9*, and *SPL15*, which are related to mossing and flowering [43]. In our study, *SPL9* was upregulated at 900 m, which was consistent with the expression trend of the downstream flowering integrator *SOC1* gene.

In other words, the area at 900 m is more suitable for the survival of *M. micrantha*.Achard and Genschik (2009) summarized the molecular model of GA signal transduction; active GA degrades the ubiquitinated DELLA protein through the 26S proteasome, which is a major step to relieve the inhibition of flowering [44]. In our study, three DELLAs were downregulated at 900 m, which might lead to flower bud differentiation of *M. micrantha* at 900 m. In the transcriptome data, we identified a large number of DEGs related to photoperiodic pathways and two temperature-related pathways (temperature and vernalization pathway), which is consistent with the results of our significant analysis of ecological factors. Annual average temperature, average temperature in the hottest month, radiation temperature, and annual sunshine duration were significantly different at three altitudes. This result might imply that temperature and light are the main factors affecting flower bud differentiation of *M. micrantha*.

The abovementioned six flowering pathways have not been isolated, and various signaling pathways ultimately achieve the regulation of flower bud differentiation by regulating integrator genes [45]. In our study, we found that the expression of three *FT* homologous genes was upregulated at 900 m, which was consistent with the expression trend of upstream flowering-promoting genes that promoted flowering. However, the three *FT* homologous genes we identified do not belong to DEGs. The *SOC1* gene can integrate flowering signals from various pathways to regulate flowering time and flower morphology [46, 47]. In our study, the expression levels of the flower integrator gene *SOC1*, flower meristem identity genes *SPL*s and *LFY*, and flower organ identities *AP1*, *CAL*, and *SEP* were significantly upregulated at the 900 m in *M. micrantha*, suggesting their important roles at 900 m. Flower meristem genes are highly conserved among plants. The expression of *LFY* in *Camellia sinensis* also affects flower bud formation, and the flower meristem genes *CAL* and *AP1* were identified in *Ambrosia artemisiifolia L*. to induce flowering [48, 49].

## The putative roles of phytohormone crosstalk in *M. micrantha* flower development

In this study, we used transcriptome technology to investigate the changes of DEGs in flower bud differentiation at 200 m, 900 m, and 1300 m. KEGG pathway enrichment analysis indicated that the DEGs were significantly enriched in pathways related to phytohormone synthesis and signal transduction processes. The results revealed that DEGs were involved in the key pathways of hormone signal transduction and biosynthetic processes associated with flower bud differentiation. This enabled us to identify key genes, and further clarify the molecular mechanisms underlying flower bud differentiation in *M. micrantha*.

In sugar apple, the expression of IAA3 increased during flower transition and flower development [26]. In *M. micrantha*, IAA3 showed the highest expression at 900 m, which may play a critical role in promoting the flower differentiation of *M. micrantha* just as it does in sugar apple. GA homeostasis is achieved by tight regulation of both activating enzymes, including GA20ox and GA3ox, which catalyze GA biosynthesis, and deactivating enzymes, (GA2ox) [50]. Interestingly, GA2ox showed significantly downregulated expression and GA20ox showed upregulated expression at 900 m in *M. micrantha*, which were in agreement with the increase in the contents of GA at 900 m. The exogenous application of GA_3_ promoted the formation of flower buds in Arabidopsis by activating family genes related to flowering in the apical meristems [51]. In our study, the content of GA_3_ reached its highest at 900 m, which implied GA_3_ plays a positive role in *M. micrantha*. In Arabidopsis, *CKX*s can reduce the content of cytokinin by removing the unsaturated isopentenyl side chain of cytokinin and LOGs play a pivotal role in regulating cytokinin activity [52–54]. In our study, *CKX6* was downregulated and LOGs were up-regulated at 900 m, which were the same trend as the highest content of IP (isoamyl alkenyl adenine) at E9. This result suggested that cytokinin may play a positive role in *M. micrantha*. Some important rate limiting enzymes in ethylene biosynthesis pathway also participate in flower bud differentiation of plants, such as *RhACS3* in *Rosa hybrida* petals is highly expressed during flower opening[55]. In *M. micrantha*, *ACS* was downregulated at 900 m, and concentration of ACC reached its lowest points at 900 m, which implied the role of ethylene as a flower repressor in *M. micrantha*. Wang et al. (2019) demonstrated that ABA content was upregulated in the later stages of flower development in *Lonicera japonica* [56]. In loquat, genes of the ABA signaling pathway and ABA content were significantly upregulated after flower anthesis [57]. In our study, genes of the ABA signaling pathway and ABA content were significantly upregulated at 1300 m, indicating that ABA plays negative roles in *M. micrantha*. .

In addition to IAA, GA, cytokinin, ethylene, and ABA, BR, JA, and SA also play important roles in flower bud differentiation. The key repressors of JA signaling transduction pathway are JASMONATE-ZIM domain (JAZ) proteins, which can positively regulate the expression of *FT* through their interaction with *MYC2* and *MYC3* [58–60]. However, we just found a *MYC2* homologous gene downregulated at 900 m, which may imply that JA plays a negative role in flower differentiation in *M. micrantha*.In *Pharbitis nil*, poor-nutrition stress-induced flowering was inhibited by aminooxy acetic acid, a phenylalanine ammonialyase inhibitor, and this inhibition was almost completely reversed by SA [61]. In our study, DEGs of the SA signaling transduction pathway were significantly downregulated at 900 m. We can infer that SA may promote flowering, but there is insufficient evidence at present, which needs to be proven by future research.

Additionally, the signaling pathways related to various plant hormones do not act independently on the regulation of flowering but are achieved by changing the expression levels of key flower-forming genes after pooling different hormone signals [62]. In summary, the combined action of various phytohormone signaling pathways affects flower bud differentiation.

In summary, nine independent cDNA libraries from *M. micrantha* flower buds at 200 m, 900 m, and 1300 m were constructed and sequenced. A large number of DEGs were identified in *M. micrantha*. Flowering time-associated and flower development-related genes were characterized based on GO and KEGG. Additionally, the expression levels of DEGs regulated to hormone signal transduction at different altitudes were also analyzed. The identification and analyses of these hormone-related genes will aid us in elucidating the regulatory mechanisms of hormones.

## Materials and Methods

### Plant material

Inflorescences and florets were taken as the investigation objects. A quadrat of 20 cm × 20 cm was randomly set for each elevation (200 m, 400 m, 800 m, 900 m, 1000 m, 1300 m) to investigate the number of inflorescences and florets in the quadrat. There were three quadrats of 20 cm × 20 cm at each elevation, with three replicates. All flower buds of *M. micrantha* for transcriptome analysis were collected from three different altitudes: (1) E2, the area at 200 m altitude (2020/10/06); (2) E9, the area at 900 m altitude (2020/10/06); and (3) E13, the area at 1300 m altitude (2020/10/06) from Tongbiguan Nature Reserve, Dehong Dai and Jingpo Autonomous Prefecture, Yunnan Province, China. The nature reserve has a subtropical monsoon climate with sufficient light and heat, abundant rainfall and a short frost period. During flower bud sample collection, flower buds from at least ten plants were mixed and regarded as one biological replicate representing each altitude, and three independent replicates were performed. Through the observation of paraffin sections at three altitudes, we found that the flower buds of *M. micrantha* collected at different altitudes are in the stage of inflorescence primordium (Additional file 8). The top of the growth point is gradually concave to form the inflorescence primordium, and the two sides form small protrusions to gradually develop into the bract primordium. Flower bud materials were frozen in liquid nitrogen immediately after collection and then stored at -80 °C until RNA was extracted. 

## Extraction of ecological factors

By using wheatA (http://www.wheata.cn/), eight eco-environmental factors were extracted from the three altitude areas of the samples collected from 2017 to 2021 for five consecutive years, including.

annual average temperature (℃), average temperature in the hottest month (℃), radiation temperature (℃), annual sunshine duration (h), relative humidity (%), evapotranspiration (mm), annual precipitation (mm), and storm runoff (mm). Based on the ANOVA, the values of the ecological factors of the three altitudes were significantly analyzed.

## Transcriptome sequencing

### RNA extraction and construction of the Illumina library

The floral buds after removing visible leaves and shoot apex from E2, E9, and E13 were used for RNA-seq. Total RNA was extracted individually using the TRIzo Kit (Promega, Beijing, China). The quantity and quality were determined using RNase-free agarose gel electrophoresis, and the total RNA concentration was measured using Agilent 2100 Bioanalyzer (Agilent Technologies, CA, USA) at 260 nm and 280 nm. Poly (A) mRNA was isolated from total RNA samples with Magnetic Oligo (dT) Beads and used for mRNA-sequencing library construction. The construction of the cDNA library was performed according to the method described by Yang et al. [63]. The cDNA was synthesized using the fragmented mRNA and end-repaired followed by a single ‘A’ base addition. An mRNA-Sequencing Sample Preparation Kit (Illumina) was used to prepare the DNA fragments for ligation to the adapters. After purification, the cDNA fragments (200 ± 25 bp) were excised and retrieved. The ligation products were size fractioned by agarose gel electrophoresis, and fragments were excised for PCR amplification.

### Sequencing, assembly and functional annotation

The mRNA-sequencing libraries were sequenced using the Illumina HiSeqTM 2500 sequencing platform (Illumina Inc., San Diego, USA) by Wuhan MetWare Biotechnology Co., Ltd. (www.metware.cn). Forde novo assembly, reads with more than 10% N bases (bases unknown) and those containing adaptor sequences were removed. Low quality reads containing more than 50% low Q (≤ 20) bases were also removed. Then, the clean reads were assembled using the Trinity program to construct unique consensus sequences [64]. The raw sequence data have been submitted to the NCBI Short Read Archive with accession numbers. The assembled unigenes were aligned to a series of protein databases using the BLASTX alignment algorithm with E < 0.00001. Annotation of the assembled unigenes was performed by searching against the NCBI Nr (nonredundant) protein (http://www.ncbi.nlm.nih.gov), Swiss-Prot (http://www.expasy.ch/sprot), KEGG (https://www.kegg.jp/kegg/kegg1.html)], KOG (http://www.ncbi.nlm.nih.gov/KOG), Pfam and TAIR (https://www.arabidopsis.org/) databases with an E-value ≤ 10^–5^ for functional annotation. Sequences were aligned to the KEGG database, significant enrichment analysis was performed, and the pathways with Q values ≤ 0.05 were defined as showing significant enrichment in DEGs. Blast2GO software was used to perform Gene Ontology (GO) term (http://www.geneontology.org) analysis. When the results conflicted among databases, the following order of priority was employed: NR, Swiss-Prot, KEGG, and KOG.

## Measurements of Plant Hormone Content

For the determination of endogenous hormones in the *M. micrantha* buds at different altitudes, individual samples from different altitudes of *M. micrantha* flower buds were harvested, immediately frozen in liquid nitrogen and stored at -80 ℃ until extraction. The samples from different altitudes used to determine endogenous hormone contents are consistent with the samples from different altitudes used for RNA sequencing. First, 50 mg of sample was ground into fine powered in liquid nitrogen, which was extracted with methanol/water/formic acid (15: 4: 1, V/V/V). The extracts from three replicates of each treatment were then combined and evaporated to dryness under a nitrogen gas stream, reconstituted in 80% methanol (V/V), and filtrated (PTFE, 0.22 μm; Anpel) before LC‒MS/MS analysis. The following determination was performed on the AB Sciex QTRAP 6500 LC‒MS/MS platform with an electrospray ESI (Agilent Technologies). An aliquot (20 μL) was injected into a Zobax XDB C18 column (2.1 mm × 100 mm × 1.8 μm, Agilent Technologies) with a flow rate of 0.35 mL/min. The mobile phase was 0.05% formic acid in water (A) and 0.05% formic acid in acetonitrile (B). The gradient was as follows: 0.0 min, A/B (95: 5, V/V); 1.0 min, A/B (95: 5, V/V); 8.0 min, A/B (5: 95, V/V); 9.0 min, A/B (5: 95, V/V); 9.1 min, A/B (95: 5, V/V); 12 min, A/B (95: 5, V/V). We performed three replicates for each assay.

## Statistical analyses

For quantification analysis of RNA-Seq, the number of fragments per kilobase of transcript per million fragments mapped (FPKM) was used to measure the expression levels of unigenes [65]. Significant differences in the contents of hormones were calculated using a one-way ANOVA with a Tukey test and a significance level at α = 0.05 in SPSS software. All expression analyses were performed in three replicates. Reported values represent arithmetic averages of three replicates. Data are expressed as the mean plus or minus standard deviation (mean ± SD). Based on the CDS of the *M. micrantha* genome, the mapping file was constructed using the annotation website (https://www.plabipd.de/portal/mercator-sequence-annotation). The expression levels of DEGs in the comparison groups of E9-vs-E2 and E9-vs-E13 were uploaded. Then, we chose PageMan functional analysis with MapMan software to annotate the biological functions of the differentially expressed genes between each sample. The WGCNA v1.48 package was used to construct a gene coexpression network. In this study, the expression levels of nine transcriptome samples (three altitudes, three repetitions each) were selected for WGCNA. R with the WGCNA package was used to build a coexpression network; the parameters of WGCNA used default settings, except that the power was 5, TOMType was unsigned, minModuleSize was 30, and mergeCutHeight was 0.25 [30]. To study the highly correlated modules, the Pearson correlation coefficient between the sample matrix and the gene module was calculated and statistically tested. The larger the correlation coefficient is, the higher the correlation between the module and the sample.

## Conclusion

In this study, comparative transcriptome analysis showed that there were significant differences in the expression of flowering pathway related DEGs and hormone signal transduction pathway‒related DEGs in the flower bud differentiation of *M. micrantha* at different altitudes. Generally, many DEGs were annotated as genes related to hormone biosynthesis, transport, or signal transduction at flower bud differentiation. Therefore, these plant hormones can be used as a control measure to protect forests and other land from *M. micrantha* invasion. Additionally, by considering six different flowering pathways, we analyzed and predicted the functions of these DEGs. Among them, key flower integrator genes and flower organ identity genes were significantly upregulated at 900 m. Flower bud differentiation in *M. micrantha* is regulated by a large number of genes involved in complex molecular pathways. These results play a critical role in illuminating the molecular mechanisms of flower bud differentiation in *M. micrantha* and will provide abundant candidate genes for studies of prevention, control, and flower-time regulation in *M. micrantha*.

## Supplementary Information

**Additional file 1: Figure S1.** KEGG annotation analysis of the most highly expressed genes in the volcano map.

**Additional file 2: Figure S2.** The GO enrichment analyses. (a) E2 vs E9 GO enrichment analyses of DEGs. (b) E13 vs E9 GO enrichment analyses of DEGs.

**Additional file 3: Table S1.** E2 vs E9 KEGG pathway enrichment.

**Additional file 4: Table S2.** E13 vs E9 KEGG pathway enrichment.

**Additional file 5: Table S3.** Differentially expressed genes (DEGs) involved in flowering-related pathways.

**Additional file 6: Table S4.** Differentially expressed genes (DEGs) involved in the signaling pathways of various hormones.

**Additional file 7: Figure S3.** Column chart of classification results annotated by the darkmagenta module from WGCNA.

**Additional file 8: Figure S4.** Morphological characteristics of flower buds of M. micrantha at three altitudes.

## Data Availability

All data generated or analyzed during this study are included in this published article and its supplementary information files. The nucleotide sequences of raw data from this study were submitted to the NCBI Sequence Read Archive (SRA) under the accession number PRJNA792910.
