# Supplementary material for: Transcriptome analysis of critical genes related to flowering in Mikania micrantha at different altitudes provides insights for a potential control
Source: BMC Genomics. 2023 Jan 10;24:14. doi: 10.1186/s12864-023-09108-8 (PMC9832669; doi:10.1186/s12864-023-09108-8)
Supplement: Supplementary file 1 — Additional file 1: Figure S1. KEGG annotation analysis of the most highly expressed genes in the volcano map. [file 12864_2023_9108_MOESM1_ESM.pdf]

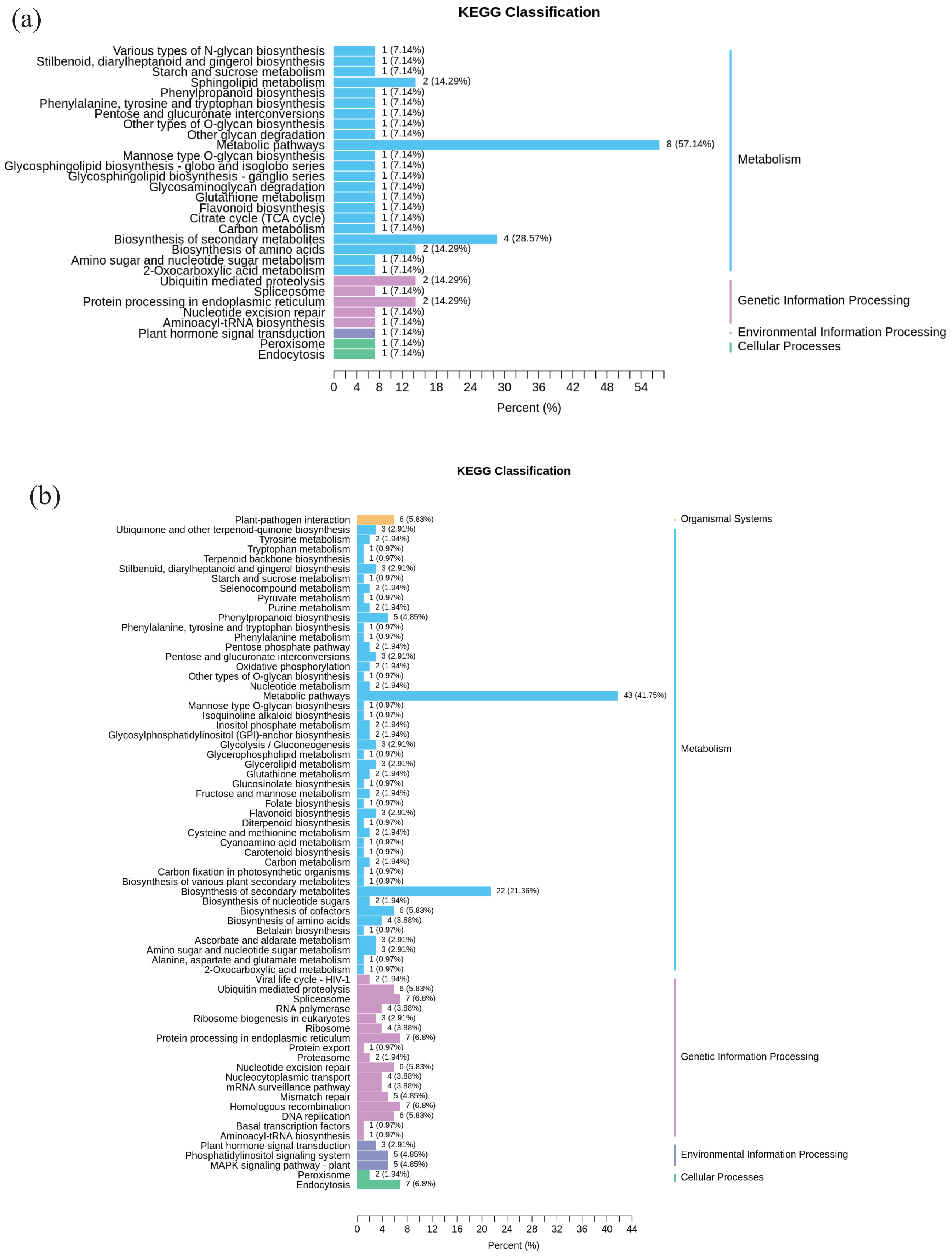

Figure S1. KEGG annotation analysis of the most highly expressed genes in the volcano map. (a) E2 vs E9 KEGG annotation analysis of the most highly expressed genes in the volcano map. (b) E13 vs E9 KEGG annotation analysis of the most highly expressed genes in the volcano map.
