# Supplementary material for: Transcriptome analysis of critical genes related to flowering in Mikania micrantha at different altitudes provides insights for a potential control
Source: BMC Genomics. 2023 Jan 10;24:14. doi: 10.1186/s12864-023-09108-8 (PMC9832669; doi:10.1186/s12864-023-09108-8)
Supplement: Supplementary file 2 — Additional file 2: Figure S2. The GO enrichment analyses. (a) E2 vs E9 GO enrichment analyses of DEGs. (b) E13 vs E9 GO enrichment analyses of DEGs. [file 12864_2023_9108_MOESM2_ESM.pdf]

(a)

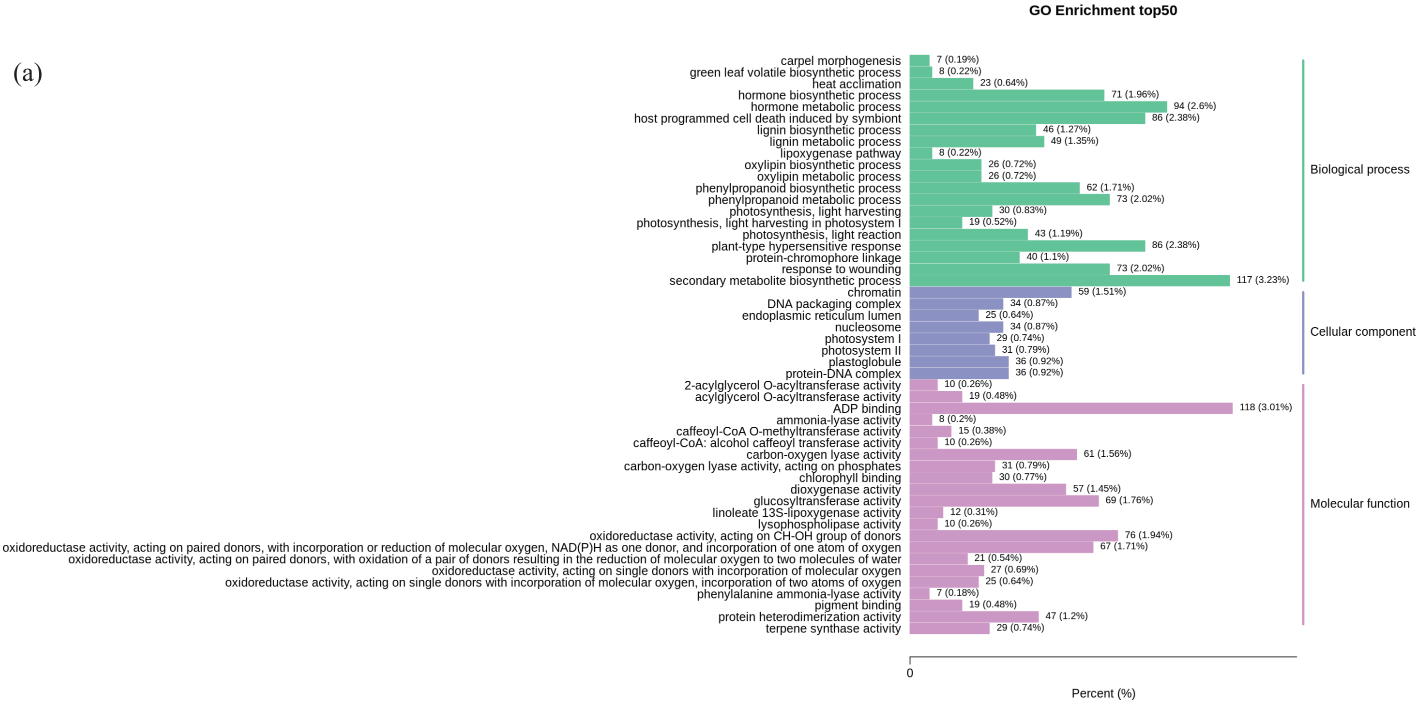

(b)

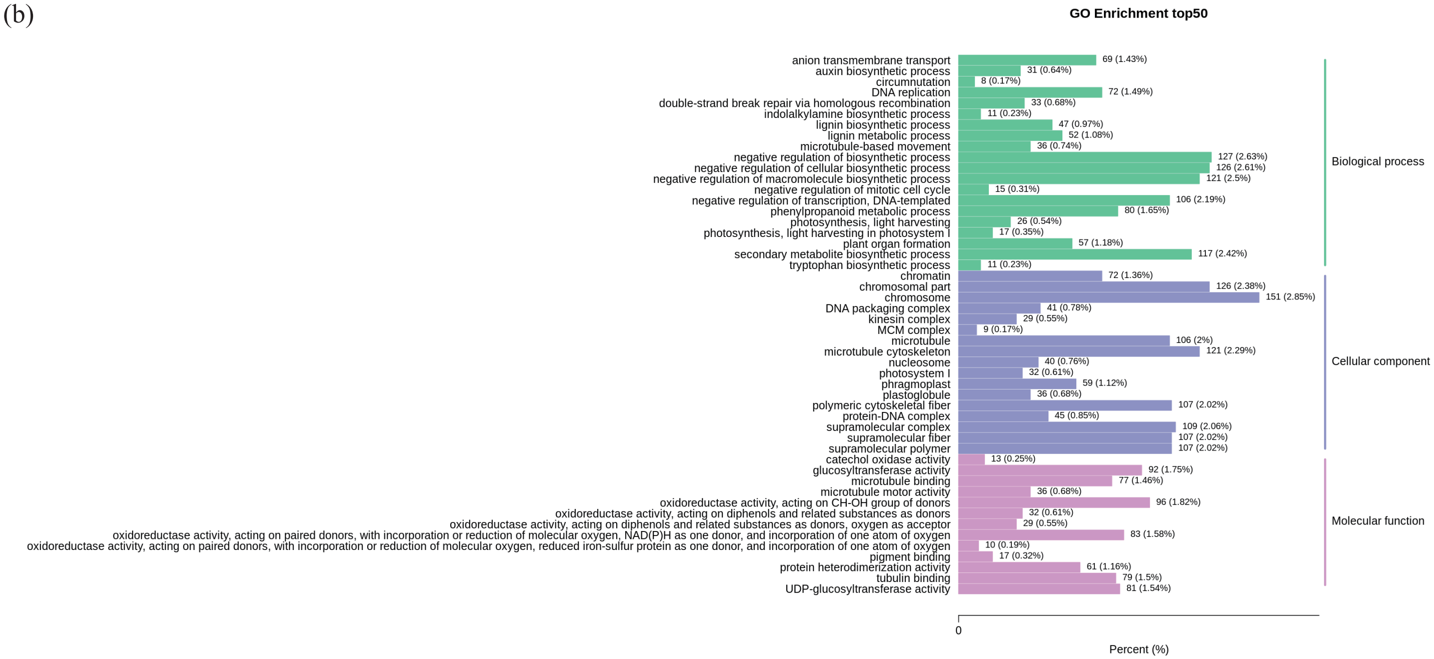

Figure S2. The GO enrichment analyses. (a) E2 vs E9 GO enrichment analyses of DEGs. (b) E13 vs E9 GO enrichment analyses of DEGs
