# Supplementary material for: Transcriptome analysis of critical genes related to flowering in Mikania micrantha at different altitudes provides insights for a potential control
Source: BMC Genomics. 2023 Jan 10;24:14. doi: 10.1186/s12864-023-09108-8 (PMC9832669; doi:10.1186/s12864-023-09108-8)
Supplement: Supplementary file 7 — Additional file 7: Figure S3. Column chart of classification results annotated by the darkmagenta module from WGCNA. [file 12864_2023_9108_MOESM7_ESM.pdf]

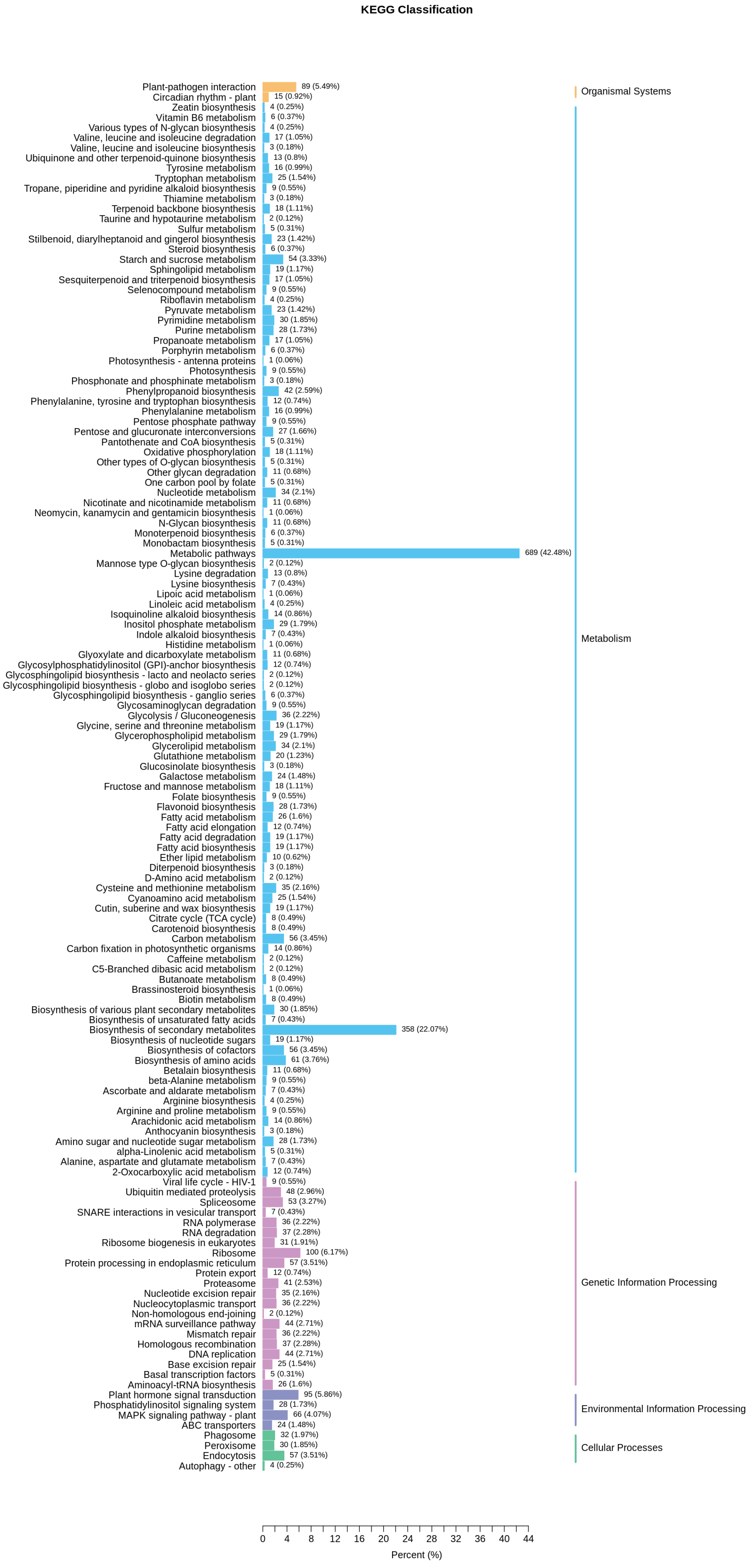

Figure S3 Column chart of classification results annotated by the darkmagenta module from WGCNA.
