# Supplementary material for: Transcriptome analysis of critical genes related to flowering in Mikania micrantha at different altitudes provides insights for a potential control
Source: BMC Genomics. 2023 Jan 10;24:14. doi: 10.1186/s12864-023-09108-8 (PMC9832669; doi:10.1186/s12864-023-09108-8)
Supplement: Supplementary file 8 — Additional file 8: Figure S4. Morphological characteristics of flower buds of M. micrantha at three altitudes. [file 12864_2023_9108_MOESM8_ESM.pdf]

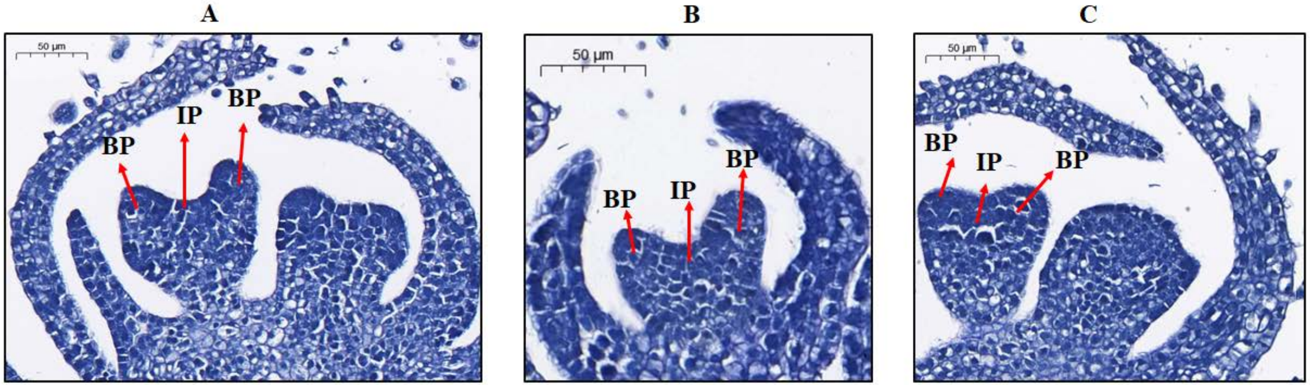

Figure S4. Morphological characteristics of flower buds of *M. micrantha* at three altitudes. (A) Morphological characteristics of flower buds of *M. micrantha* at E2; (B) Morphological characteristics of flower buds of M. *micrantha* at E9; (C) Morphological characteristics of flower buds of *M. micrantha* at E13; bar=50 µm; IP: Inflorescence primordium; BR: Bracts primordium.
